# The Dual-Specificity Kinase DYRK1A Modulates the Levels of Cyclin L2 To Control HIV Replication in Macrophages

**DOI:** 10.1128/JVI.01583-19

**Published:** 2020-02-28

**Authors:** Javan K. Kisaka, Lee Ratner, George B. Kyei

**Affiliations:** a Department of Medicine, Washington University School of Medicine in St. Louis, St. Louis, Missouri, USA; b Department of Molecular Microbiology, Washington University School of Medicine in St. Louis, St. Louis, Missouri, USA; c Department of Virology, Noguchi Memorial Institute for Medical Research, College of Health Sciences, University of Ghana, Accra, Ghana; Emory University

**Keywords:** DYRK1A, cyclin L2, human immunodeficiency virus, macrophages, protein phosphorylation

## Abstract

HIV replication in macrophages contributes to the latent viral reservoirs, which are considered the main barrier to HIV eradication. Few cellular factors that facilitate HIV replication in latently infected cells are known. We previously identified cyclin L2 as a critical factor required by HIV-1 and found that depletion of cyclin L2 attenuates HIV-1 replication in macrophages. Here we demonstrate that cyclin L2 promotes HIV-1 replication through interactions with the dual-specificity tyrosine phosphorylation-regulated kinase 1A (DYRK1A). Cyclin L2 and DYRK1A were colocalized in the nucleus and were found together in immunoprecipitation experiments. Knockdown or inhibition of DYRK1A increased HIV-1 replication in macrophages, while depletion of cyclin L2 decreased HIV-1 replication. Furthermore, depletion of DYRK1A increased expression levels of cyclin L2. DYRK1A is a proline-directed kinase that phosphorylates cyclin L2 at serine residues. Mutations of cyclin L2 at serine residues preceding proline significantly stabilized cyclin L2 and increased HIV-1 replication in macrophages. Thus, we propose that DYRK1A controls cyclin L2 expression, leading to restriction of HIV replication in macrophages.

**IMPORTANCE** HIV continues to be a major public health problem worldwide, with over 36 million people living with the virus. Although antiretroviral therapy (ART) can control the virus, it does not provide cure. The virus hides in the genomes of long-lived cells, such as resting CD4^+^ T cells and differentiated macrophages. To get a cure for HIV, it is important to identify and characterize the cellular factors that control HIV multiplication in these reservoir cells. Previous work showed that cyclin L2 is required for HIV replication in macrophages. However, how cyclin L2 is regulated in macrophages is unknown. Here we show that the protein DYRK1A interacts with and phosphorylates cyclin L2. Phosphorylation makes cyclin L2 amenable to cellular degradation, leading to restriction of HIV replication in macrophages.

## Abstract (precis)

HIV continues to be a major public health problem worldwide, with over 36 million people living with the virus. Although antiretroviral therapy (ART) can control the virus, it does not provide cure. The virus hides in the genomes of long-lived cells, such as resting CD4^+^ T cells and differentiated macrophages. To get a cure for HIV, it is important to identify and characterize the cellular factors that control HIV multiplication in these reservoir cells. Previous work showed that cyclin L2 is required for HIV replication in macrophages. However, how cyclin L2 is regulated in macrophages is unknown. Here we show that the protein DYRK1A interacts with and phosphorylates cyclin L2. Phosphorylation makes cyclin L2 amenable to cellular degradation, leading to restriction of HIV replication in macrophages.

## INTRODUCTION

Infection with human immunodeficiency virus (HIV) continues to be a public health challenge, with over 1.2 million deaths annually (1). Although antiretroviral therapy (ART) can suppress the virus and reduces mortality, it does not provide cure (2, 3). The main obstacle to an HIV cure is persistence of the provirus in latently infected quiescent cells, such as resting CD4^+^ T cells (4–8). HIV latency may be established through infection of actively dividing cells that revert to a resting state (9, 10) or direct infection of resting CD4^+^ T cells (11, 12). Other potential reservoirs include macrophages and microglia in the central nervous system (13–16). Although the contribution of macrophages to the HIV reservoir is still debated, accumulating evidence suggests that they could play an important role (17). First, the discovery of long-lived yolk sac-derived tissue resident macrophages capable of self-renewal provides a new paradigm for viewing macrophages as potential reservoirs. Second, Honeycutt et al. have shown in a macrophage-only mouse model that a form of latency and reactivation after ART interruption is feasible (18). Third, some virologically suppressed macaques on ART can express simian immunodeficiency virus (SIV) in brain macrophages when treated with latency-reversing agents (19). Fourth, resident macrophages in the urethras of patients suppressed on ART have integrated HIV DNA, can produce RNA, and can be reactivated to make replication-competent virus (20). Identification and targeting of cellular factors that control HIV-1 replication in quiescent cells constitute an important intervention strategy toward HIV-1 eradication. Therefore, there is a need to identify more cellular factors that control HIV replication, transcription, and reactivation in macrophages.

A group of proteins that are becoming increasingly important in the HIV life cycle are cyclins and their counterparts cyclin-dependent kinases (CDKs). Cyclins are proteins that regulate the cell cycle. They are synthesized during interphase and rapidly degraded at each mitotic phase (21, 22). They bind to and activate specific CDKs which, in turn, control cell division and transcription (23–25). Cyclins determine the activity, cellular localization, substrate specificity, and stability of the CDK (22, 26). Several cyclins have been shown to play a role in different parts of the HIV life cycle. The most important and well-characterized of these is cyclin T1/CDK9 (P-TEFb), a complex required for HIV transcription through interaction with Tat (27). In macrophages, as in resting T cells, the levels of cyclin T are reduced, and this may play a role in latency (27). Recently, CDK11, which partners with cyclin L, was shown to regulate HIV 3′ mRNA processing. Cyclin L interacts with CDK11, which phosphorylates the carboxyl-terminal domain (CTD) of polymerase II (Pol II) and splicing factor SC35. Phosphorylation of the CTD is required for transcription initiation, elongation, and RNA processing (28–31). The cyclin L family includes cyclins L1 and L2. Cyclin L1 has three isoforms, L1α, L1β, and L1γ, whereas cyclin L2 has two isoforms, L2α and L2β (32, 33). Loyer et al. determined that both cyclins L1 and L2 are ubiquitously expressed in human cell lines and mouse tissues and that both interact with essential splicing factors (33).

Using a yeast two-hybrid screen, we previously identified cyclin L2 as a critical factor required for HIV replication in noncycling cells, such as differentiated THP-1 cells and monocyte-derived macrophages (MDMs), but not in dividing cells (34). Cyclin L2 binds to and induces degradation of HIV-1 restriction factor sterile alpha motif and HD domain-containing protein 1 (SAMHD1) by recruiting cellular factors DCAF1, Cul4, and DDB1 to the proteasome. The depletion of cyclin L2 abrogates SAMHD1 degradation and decreases HIV-1 replication in macrophages (34). Cyclin L2 has an N-terminal RNA-binding domain (the “cyclin box”) common for all cyclins and a C-terminal arginine- and serine-rich (RS) domain present only in the cyclin L proteins (35). The RS domain-containing proteins are essential splicing factors that are associated with the spliceosome (36). The RS domain is rich in dipeptide repeats of arginine and serine residues (30). It enables the cyclins to localize to nuclear speckles (37, 38), where they are thought to be involved in pre-mRNA processing (33, 37, 39).

Cyclin L2 is phosphorylated by the dual-specificity tyrosine phosphorylation-regulated kinase 1A (DYRK1A) (39). DYRK1A belongs to the DYRK family of protein kinases (40). In mammals, there are five subclasses (DYRK1A, DYKRK1B, DYRK2, DYRK3, and DYRK4) (40, 41). They share a conserved catalytic domain, but only DYRK1A has a C-terminal domain rich in serine and threonine residues. DYRK1A is expressed in the nucleus (42, 43) and the cytosol (41, 44). In the nucleus, it is localized in the nuclear speckles, suspected to be splicing factor compartments (43, 45). DYRK1A has been associated with many cellular processes, such as brain development, T cell differentiation, and pancreatic islet cell formation (41, 46, 47). de Graaf and colleagues identified DYRK1A as a putative kinase for cyclin L2. Overexpression of DYRK1A in COS-7 cells increased the phosphorylation of cyclin L2 (39). Since most cyclins are controlled by phosphorylation (23), we postulated that the effect of cyclin L2 on HIV may be regulated through interactions with DYRK1A. Here we show that cyclin L2 interacts with DYRK1A in cycling and noncycling cells. Knockdown or pharmacological inhibition of DYRK1A resulted in a severalfold increase in HIV replication in nondividing cells but had minimal effect in dividing cells. This increase in HIV replication upon DYRK1A inhibition is dependent on intact cyclin L2. We found that depletion of DYRK1A increases cyclin L2 levels, thus increasing HIV replication. We present evidence to show that DYRK1A enhances the degradation of cyclin L2 through phosphorylation to restrict HIV replication in macrophages.

## RESULTS

### Cyclin L2 interacts with DYRKIA through its RS domain.

**FIG 1:**
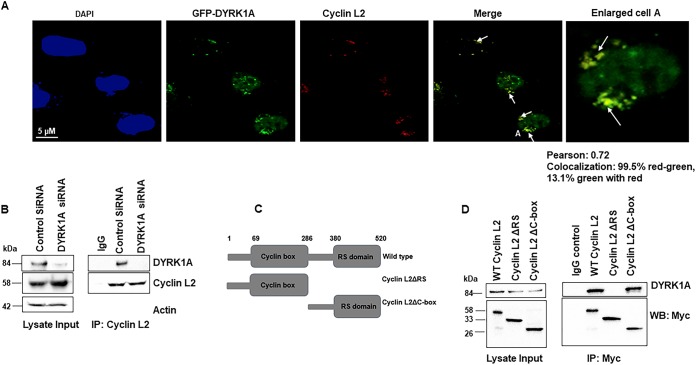
Cyclin L2 interacts with DYRK1A through the RS domain. (A) Cyclin L2 colocalizes with DYRK1A in the nucleus. HeLa cells were transfected with GFP-DYRK1A and immunostained for endogenous cyclin L2 (red). Cell A is enlarged to highlight colocalization. Arrows show areas of colocalization. Percent colocalization is shown by Pearson correlation. (B) Endogenous cyclin L2 coimmuniprecipitates with DYRK1A. Cyclin L2 antibody or IgG was used to immunoprecipitate the protein in differentiated THP-1 cells transfected with control or DYRK1A siRNA and immunoblotted for the endogenous proteins. (C) Schematic of the cyclin L2 constructs used for experiments whose results are shown in panel D. (D) The RS domain of cyclin L2 is required for interaction with DYRK1A. 293T cells were transfected with the indicated Myc-tagged cyclin L2 constructs in panel C and immunoprecipitation was performed with monoclonal Myc antibody. Western blots were probed for Myc or endogenous DYRK1A. Blots are representative of results from two or three independent experiments.

To determine whether cyclin L2 interacts with DYRK1A, we transfected HeLa cells with green fluorescent protein (GFP)-DYRK1A and immunostained for endogenous cyclin L2. As shown in Fig. 1A, most of the cyclin L2 in the nucleus colocalized with DYRK1A in nuclear speckles (39). We confirmed these findings in macrophage-like cells by performing coimmunoprecipitations for the two endogenous proteins in differentiated THP-1 cells. Figure 1B shows that cyclin L2 coimmunoprecipitated with DYRK1A in cells transfected with control small interfering RNA (siRNA) but not in DYRK1A knockdown cells or IgG controls. These results show that the two proteins specifically interact. Next, we determined which domain of cyclin L2 is responsible for the interaction. Cyclin L2 has two main domains, the cyclin box in the N terminus and an arginine- and serine-rich (RS) domain in the C terminus (Fig. 1C). To find which domain is critical for interacting with DYRK1A, we constructed three Myc-tagged clones consisting of the full-length cyclin L2, cyclin L2ΔRS (residues 1 to 286), and cyclin L2Δc-box (residues 287 to 520). We transfected 293T cells with these constructs, pulled down Myc with a monoclonal antibody, and immunoblotted for endogenous DYRK1A. Full-length cyclin L2 interacted with DYRK1A, as expected (Fig. 1D). In addition, the mutant containing the RS domain (cyclin L2Δc-box) coimmunoprecipitated with DYRK1A. However, the mutant lacking the RS domain (cyclin L2ΔRS) could not pull down DYRK1A, indicating that the RS domain is required for the interaction between the two proteins.

### Knockdown of DYRK1A disproportionately increases HIV replication in nondividing cells.

**FIG 2:**
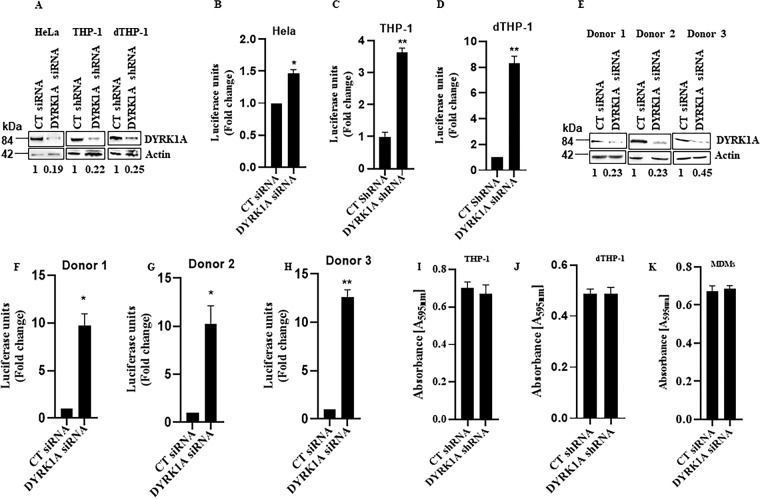
Knockdown of DYRK1A disproportionately increases HIV replication in nondividing cells. (A) Western blot showing the knockdown of DYRK1A in HeLa, THP-1, and differentiated THP-1 (dTHP-1) cells. Cell lysates taken at 48 h prior to viral infections are shown for each cell type. (B) HeLa cells were transfected with control or DYRK1A siRNA for 48 h and infected with VSV-G-pseudotyped HIV-1Luc for 48 h. (C and D) Stable THP-1 cells expressing control or DYRK1A shRNA were either directly infected with HIVLuc or differentiated into macrophages and then infected with HIVLuc for 48 h. HIV infection was measured by luciferase luminescence in cell lysates normalized to total protein concentration. (E) Knockdown DYRK1A in monocyte-derived macrophages (MDMs) isolated from HIV-negative donors after 48 h of siRNA transfection. (F to H) MDMs from three different donors were infected with VSV-G-pseudotyped HIV-1Luc for 48 h and HIV infection was measured as described above. (I and J) Differentiated THP-1 cells with control of DYRK1A shRNA were infected with HIV-1 for 48 h and the MTT assay was performed as described in Materials and Methods. (K) MDMs with control or DYRK1A siRNA were infected with HIV-1 for 48 h and the MTT assay was performed. Data are means, and error bars indicate SEM (*n* = 3). **, *P* < 0.001; *, *P* < 0.05, Student’s *t* test.

**FIG 3:**
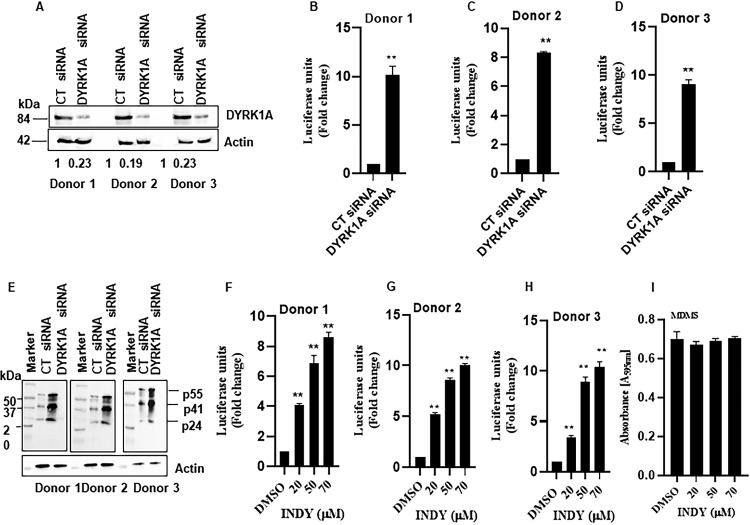
Knockdown or pharmacological inhibition of DYRK1A increases HIV replication in multiple rounds of infection. (A) Western blots showing DYRK1A knockdown in three HIV-negative donors. (B to D) MDMs with control of DYRK1A siRNA were infected with macrophage-tropic replication-competent HIV-1 (BaL-3) for 6 h and washed, and medium was replaced after 48 h. Viral particles were collected after 72 h and used to transduce TZM-bl indicator cells. Luciferase luminescence in cell lysates was used as a measure of HIV replication. (E) Cell lysates from the respective MDMs were used for Western blots for HIV-1 Gag and actin. (F to H) MDMs were infected with HIV-1 BaL-3 for 72 h in the presence of INDY or dimethyl sulfoxide (DMSO). HIV replication was measured as for panels B to D. (I) MDMs were treated with INDY and infected with HIV-1 for 48 h, and the MTT assay was performed as described in Materials and Methods. Data are means, and error bars indicate SEM (*n* = 3). *, *P* < 0.01; **, *P* < 0.0001 (Student’s *t* test).

In dividing cells, previous work showed that depletion of DYRK1A results in less than a 2-fold increase in HIV replication (48). Given that the action of cyclin L2 on HIV replication is restricted to macrophages and the two proteins interact, we wondered what effect DYRK1A would have on HIV replication in nondividing cells. Therefore, we knocked down DYRK1A in HeLa and THP-1 monocytic cells to compare degrees of HIV replication in the two cell types (Fig. 2A). As previously reported (48), there was about a 1.5-fold increase in HIV infection in HeLa cells (Fig. 2B). To test the idea that DYRK1A may be more important for HIV replication in monocytes/macrophages, we used stable THP-1 cells carrying either control or DYRK1A small hairpin RNA (shRNA). Since THP-1 cells can be differentiated into macrophage-like cells with phorbol 12-myristate 13-acetate (PMA), they provide a good model to compare the roles of DYRK1A in HIV infection in dividing and nondividing cells. Knockdown of DYRK1A with shRNA in undifferentiated THP-1 monocytes resulted in about a 3-fold increase in HIV infection (Fig. 2C). However, when the THP-1 cells were differentiated (dTHP-1) before infection, HIV replication increased to 8-fold (Fig. 2D). To confirm these results, we used siRNA to knock down DYRK1A in primary monocyte-derived macrophages (MDMs) isolated from three different HIV-negative donors (Fig. 2E). As shown in Fig. 2F to H, in DYRK1A knockdown cells, HIV infection increased 9.7- to 12.7-fold depending on the donor. The increases in HIV replication were not due to proliferation of THP-1 cells or MDMs, as shown by the cell proliferation assays whose results are shown in Fig. 2I to K. Next, we used macrophage-tropic replication-competent HIV-1 (BaL-3) to determine if DYRK1A would have the same effect with multiple rounds of infection. We infected control or DYRK1A knockdown MDMs isolated from three different donors (Fig. 3A) with HIV-1 BaL-3 for a total of 72 h. After 24 h of infection, we replaced the media and collected supernatants after 48 h. We then used the collected virus to infect TZM-bl indicator cells. As shown in Fig. 3B to D, knockdown of DYRK1A increased replication of HIV-1 8- to 10-fold depending on the donor. Cell lysates from the MDMs were processed for Western blotting and showed a significant increase in HIV Gag proteins in DYRK1A knockdown cells (Fig. 3E). Finally, we employed INDY, an established inhibitor of DYRK1A which binds to the protein’s ATP pocket to inhibit its action (49). Treatment with INDY increased HIV replication in a dose-dependent manner in all three donors (Fig. 3F to H). Treatment with INDY did not result in proliferation of MDMs (Fig. 3H). Taken together, these results confirm that DYRK1A has a more pronounced effect on HIV replication in macrophages.

### Depletion of cyclin L2 abolishes DYRK1A-mediated HIV restriction.

**FIG 4:**
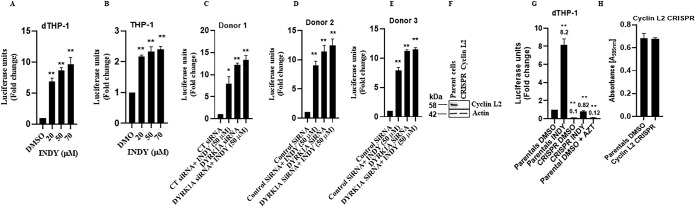
Depletion of cyclin L2 abolishes DYRK1A-mediated HIV restriction. (A) Differentiated THP-1 cells were infected with VSV-G-pseudotyped HIV-1Luc for 48 h in the presence of DMSO or increasing concentrations of INDY. Luciferase luminescence in cell lysates was used as a measure of HIV infection. (B) Undifferentiated THP-1 cells were infected with VSG-G-pseudotyped HIV-1Luc for 48 h with or without INDY. HIV infection was measured as described above. (C to E) Monocyte-derived macrophages were transfected with control or DYRK1A siRNA for 48 h. Cells were infected with VSV-G-pseudotyped HIV-1Luc for 48 h and HIV infection was measured as described above. (F) Western blot showing cyclin L2 CRISPR/Cas9 knockout in differentiated THP-1 cells. (G) Cyclin L2 CRISPR/Cas9 knockout differentiated THP-1 cells or parental controls were infected with HIV-1Luc with or without INDY for 48 h. HIV nonnucleoside reverse transcriptase inhibitor zidovudine (AZT; 20 μM) was used as a positive control. (H) Parental or cyclin L2 knockout THP-1 cells were infected with HIV-1 for 48 h and the MTT assay was performed. Data are means, and error bars indicate SEM (*n* = 3). *, *P* < 0.001; **, *P* < 0.0001 (Student’s *t* test).

Since cyclin L2 promotes HIV replication in macrophages and DYRK1A has the opposite effect, we investigated whether the DYRK1A restriction of HIV replication in macrophages is dependent on intact cyclin L2. If that were the case, depletion of cyclin L2 would abolish the effect of DYRK1A illustrated in Fig. 2 and 3. Consistent with the DYRK1A knockdown results, treatment of differentiated THP-1 cells with INDY increased HIV infection up to 10-fold (Fig. 4A), compared to only 2-fold in undifferentiated cells (Fig. 4B). To show that INDY worked through DYRK1A, we repeated the experiments with MDMs from three donors. When INDY (50 μM) was added to the MDMs with DYRK1A knockdown, no further increase in HIV replication was observed, indicating that the effect of INDY on HIV replication was likely mediated through DYRK1A (Fig. 4C to E). Next, we used cyclin L2 CRISPR/Cas9 knockout THP-1 cells to interrogate the effect of DYRK1A inhibition in the context of cyclin L2 depletion. In control parental cells, treatment with INDY increased HIV infection 8-fold. However, in cyclin L2 knockout cells, the effect of INDY was reduced only 0.8-fold (Fig. 4F and G), without a decrease in cell numbers (Fig. 4H). This shows that the interaction between the two proteins has functional consequences on HIV replication and that intact cyclin L2 is required for the effect of DYRK1A on HIV replication.

### DYRK1A controls cyclin L2 levels in macrophages.

**FIG 5:**
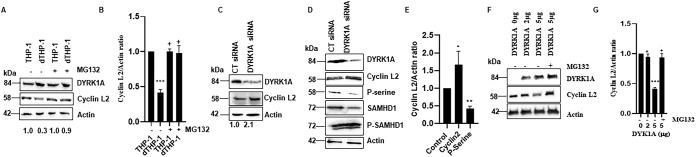
Knockdown of DYRK1A increases levels of cyclin L2, while overexpression of DYRK1A reduces cyclin L2 levels. (A) Western blot showing levels of endogenous cyclin L2 and DYRK1A in THP-1 and differentiated THP-1 cells with and without the proteasome inhibitor MG132. Results represent those from three independent experiments. Levels of cyclin L2 normalized to actin are shown below the blot. (B) Quantitation of cyclin L2 expression levels in dividing and nondividing THP-1 cells. (C) Differentiated THP-1 cells were transfected with control or DYRK1A siRNA, and levels of cyclin L2 and DYRK1A were measured by Western blotting. The experiment was repeated three times, and cyclin L2 actin ratios are shown below the blot. (D and E) Differentiated THP-1 cells were transfected with control or DYRK1A siRNA and Western blotting was performed for the indicated proteins in the cell lysates. A phosphoserine antibody was used to detect phosphorylated cyclin L2 (P-serine) after immunoprecipitation. Quantification of total cyclin L2 and phosphorylated portion relative to actin from three experiments is shown in panel E. (F and G) Myc-DYRK1A was expressed in increasing plasmid concentrations in 293T cells for 24 h with and without MG132. Levels of endogenous cyclin L2 were detected by Western blotting and quantified for three independent experiments (G). Data are means, and error bars indicate SEM (*n* = 3). *, *P* < 0.05; **, *P* = 0.001; ***, *P* < 0.0001; ^+^, *P* > 0.05.

Next we proceeded to determine a possible mechanism for how cyclin L2-DYRK1A interactions control HIV replication in macrophages. Since DYRK1A is a kinase for cyclin L2 and multiple kinases modulate the levels of their respective cyclins (50, 51), we wondered if DYRK1A controls cyclin L2 levels in macrophages. For instance, if DYRK1A reduced the levels of cyclin L2 in macrophages, that could reduce the amount of HIV produced in those cells. First, we immunoblotted for levels of the two proteins in dividing and nondividing THP-1 cells. As shown in Fig. 5A and B, while the levels of DYRK1A were higher in differentiated THP-1 cells, cyclin L2 levels were 2.5-fold lower in the same cells. When the cells were treated with the proteasome inhibitor MG132, the levels of cyclin L2 in dTHP-1 cells were restored to the levels detected in dividing cells. This suggests that increased expression of DYRK1A in nondividing cells accelerates degradation of cyclin L2 through the proteasome. To determine a direct role for DYRK1A in modulating cyclin L2 levels, we immunoblotted for cyclin L2 in control versus DYRK1A knockdown differentiated THP-1 cells. Figure 5C shows that compared to those in controls, cyclin L2 levels increased 2-fold in cells with DYRK1A depletion. Next, we immunoprecipitated cyclin L2 in dTHP-1 cells treated with control or DYRK1A siRNA and immunoblotted for total cyclin L2 or phosphorylated cyclin L2. While total cyclin L2 was increased, the phosphorylated portion was reduced, as expected (Fig. 5D and E). In addition, total SAMHD1 levels were reduced but phosphorylated levels remained the same, consistent with our previous findings that increased cyclin L2 reduces SAMHD1 levels but has no effect on the phosphorylated portion (34). Finally, we overexpressed increasing amounts of DYRK1A to determine the effect on cyclin L2 levels. Increasing levels of DYRK1A resulted in proportional degradation of cyclin L2 (Fig. 5F and G). When MG132 was added where DYRK1A had the most effect, cyclin L2 degradation was rescued. Taken together, these data show that DYRK1A increases the turnover of cyclin L2 through the proteasome, resulting in reduced HIV production.

### Cyclin L2 is stabilized by dephosphorylation to increase HIV replication.

To test the possibility that DYRK1A-mediated phosphorylation increases cyclin L2 degradation, we mutated serine residues in cyclin L2 shown previously to be phosphorylated by DYRK1A (39). We made phospho mutants of cyclin L2 by replacing serine residues that are adjacent to proline with alanine in the RS domain at the C terminus. For mutant 1, named P-369, four serine residues were mutated to alanine: S330, S338, S348, and S369. For mutant 2, named P-330, seven serine residues were mutated to alanine: S330, S338, S348, S369, S394, S401, and S427.

**FIG 6:**
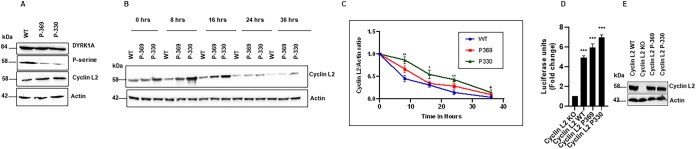
Mutation of DYRK1A phosphorylation sites increases cyclin L2 stability and HIV replication in differentiated THP-1 cells. (A) Phosphorylation of cyclin L2 decreases with increasing numbers of serine-to-alanine mutants. 293T cells were cotransfected with plasmids expressing GFP-DYRK1A and Myc-cyclin L2 mutants (cyclin L2 WT, cyclin L2 P369, and cyclin L2 P330; see text for details) for 48 h. Cells were lysed and immunoblotted with DYRK1A antibody, P-serine, and Myc. (B and C) Cyclin L2 stability is increased by phosphorylation site mutations. Cells expressing WT and mutant cyclin L2 were treated with cycloheximide to block new protein synthesis. Cells were lysed at the indicated time points and Western blotting was performed for Myc-cyclin L2 and actin. Quantification of cyclin L2/actin ratios was used to generate degradation curves from three independent experiments (C). (D) Myc-tagged mutant and wild-type cyclin L2 were expressed in cyclin L2 CRISPR/Cas9 knockout THP-1 cells, differentiated with PMA, and infected with HIV-1Luc. HIV infection was measured as indicated previously. (E) Expression of Myc-cyclin L2 WT and mutants in dTHP-1 cells used for panel D. Data are means, and error bars indicate SEM (*n* = 3). ***, *P* < 0.001, ANOVA.

First, we expressed the wild type (WT) and mutants together with DYRK1A in 293T cells and immunoblotted for phosphorylated and total cyclin L2. As shown in Fig. 6A, while the total levels of the mutants were increased compared to those of WT cyclin L2, the phosphorylated portions were reduced, as expected. This indicated that the dephosphorylated cyclin L2 mutants may be more stable than the WT. To determine the stability of the WT and mutants in a more methodical manner, we treated cells with cycloheximide to block new protein synthesis and performed a chase for 36 h to generate degradation curves. The results showed that the mutant with the most mutated DYRK1A phosphorylation sites (P-330) was the slowest to degrade (Fig. 6B and C). This shows that phosphorylation of cyclin L2 by DYRK1A increased cyclin L2 degradation. Next, we tested if the mutants were functional and whether they affected HIV-1 replication. We expressed the Myc-tagged mutants in cyclin L2 CRISPR/Cas9 knockout THP-1 cells, differentiated with PMA and infected with HIV-1 Luc. As shown in Fig. 6D, wild-type cyclin L2 retained its function in THP-1 cells. We observed a 5-fold increase in HIV replication in cells expressing wild-type cyclin L2 and up to a 7.5-fold increase in cells expressing cyclin L2 (P-330), the mutant with the most dephosphorylation. This suggests that the more stable cyclin L2 in macrophages, the higher the HIV replication.

### DYRK1A inhibition increases HIV-1 transcription.

**FIG 7:**
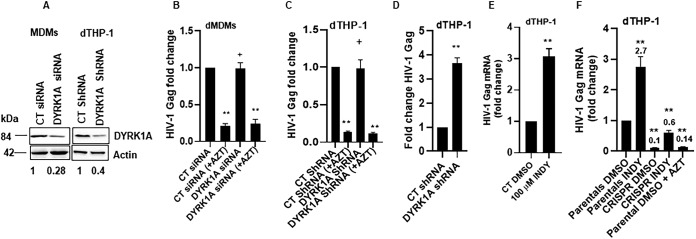
Inhibition of DYRK1A increases HIV-1 transcription. (A) Western blots showing knockdown of DYRK1A in MDMs and differentiated THP-1 cells. (B and C) MDMs or dTHP-1 cells were infected with HIV-1Luc for 24 h. To measure late reverse transcription, quantification of HIV DNA was done by qPCR. (D) Differentiated THP-1 cells with control or DYRK1A shRNA were infected with full-length HIV-1 for 24 h, washed, and incubated with raltegravir and darunavir for another 24 h. Total cell RNA was isolated and reverse transcription-quantitative PCR (qRT-PCR) was performed for *gag* mRNA as a measure of HIV transcription. (E) Differentiated THP-1 cells treated with DMSO or INDY were infected as for panel D and *gag* mRNA was quantified. (F) Cyclin L2 CRISPR/Cas9 knockout or parental control differentiated THP-1 cells were infected as for panel D and *gag* mRNA was quantified. Data are means, and error bars indicate SEM (*n* = 3). **, *P* < 0.0001; ^+^, *P* > 0.05, unpaired Student’s *t* test.

To determine which step of the viral life cycle is modulated by the cyclin L2-DYRK1A interaction, we performed experiments to evaluate late reverse transcription (RT) and transcription of HIV provirus. We infected MDMs and dTHP-1 cells with HIV-1 BaL-3 and quantified the levels of HIV-1 DNA by quantitative PCR (qPCR) 48 h postinfection to determine whether DYRK1A knockdown had any effect on late reverse transcription. As shown in Fig. 7A to C, the levels of HIV-1 *gag* DNA were unchanged, suggesting that knockdown of DYRK1A did not alter reverse transcription in either MDMs or differentiated THP-1 cells. Next, to determine HIV mRNA expression from integrated HIV provirus, we infected differentiated THP-1 cells with full-length HIV-1 for 24 h, washed and treated with integrase inhibitor raltegravir and protease inhibitor darunavir to prevent new integration and reinfection, respectively. After 24 h of INDY treatment, we isolated RNA from the cells and measured *gag* mRNA as a measure of HIV transcription. We observed a 3-fold increase in *gag* mRNA upon DYRK1A knockdown (Fig. 7D) or inhibition with INDY (Fig. 7E). Finally, to confirm that this effect on transcription was through cyclin L2, we used cyclin L2/CRISPR/Cas9 knockout cells. As shown in Fig. 7F, knockout of cyclin L2 abrogated the increase in transcription observed with DYRK1A knockdown. Taken together, these data show the requirement for cyclin L2 for DYRK1A actions on HIV-1 replication in macrophages.

## DISCUSSION

Identification of cellular factors involved in HIV-host interactions is critical to understanding mechanisms of persistence and latency, which could eventually lead to cure. Here we show that cyclin L2, previously shown to degrade SAMHD1, interacts with the kinase DYRK1A. Knockdown or pharmacological inhibition of DYRK1A had pronounced effects on HIV replication in macrophages compared to dividing cells, even with multiple rounds of infection. Overall, the data point to a mechanism whereby inhibition of DYRK1A results in increased amounts and a more stable cyclin L2 which consequently promote HIV replication in macrophages. Since cells with knockdown of both proteins produce little HIV, it is likely that cyclin L2 plays the central role in this interaction. Degradation of a cyclin by DYRK1A is not without precedent. Thompson et al. showed that DYRK1A phosphorylates cyclin D3 to promote its degradation and enhance quiescence in T and B cells (52). Degradation of cyclin L2 leads to restriction of HIV infection in macrophages but less so in dividing cells. The reason for the pronounced effect in nondividing cells is still unclear. Given that SAMHD1 phosphorylation makes it inactive in dividing cells (53, 54), it is possible that differential phosphorylation in dividing versus nondividing cells could be the ultimate mechanism. Here, in the context of a clean background (cyclin L2 knockout cells), we show that expression of cyclin L2 in macrophages increases HIV replication, confirming our previous results (34). We found that terminally differentiated cells express more DYRK1A, and less cyclin L2, which correlates with the production of less HIV. It will be interesting to determine how the interplay between the two proteins affects HIV replication in resting T cells, which are considered differentiated and critical for HIV latency. Supporting the assertion that cyclin L2/DYRK1A interactions may be critical in HIV latency are the findings that inhibitors of DYRK1A increase HIV latency reversal in resting T cells (55). Although the mechanism for this finding is not known, it will not be surprising if cyclin L2 plays a role in resting T cells similar to the one it plays in macrophages.

A unique feature of the cyclin L proteins is possession of the RS domain, a hallmark of splicing factors (33, 37, 39, 56). Cyclin L2 interacts with splicing factors and is believed to be involved in pre-mRNA splicing. Interestingly, we found that the RS domain is required for interactions with DYRK1A, making it likely that DYRK1A also localizes in splicing factor compartments. We found that knockdown of DYRK1A resulted in increased HIV mRNA production from integrated provirus. This could be at the level of transcription or splicing. Since knockdown of cyclin L2 abrogated this effect, it would suggest that cyclin L2 plays a role in HIV transcription or splicing in macrophages. Recent findings that SAMHD1 plays a role in HIV transcription in resting cells (57) make this an interesting line of investigation for future studies. Previous studies showed that DYRK1A inhibits HIV transcription in actively dividing cells through nuclear factor of activated T cells (NFAT) (48). However, how HIV transcription or splicing in macrophages is controlled by cyclin L2, DYRK1A, and NFAT interplay remains to be determined.

We found that cyclin L2 is degraded through the proteasome and that this degradation was dependent on DYRK1A. Knockdown of DYRK1A or mutations of its phosphorylation sites in cyclin L2 had the same effect of stabilizing cyclin L2 and increasing HIV replication in macrophages. In this regard, cyclin L2 resembles cyclin D1 and cyclin E, which are also stabilized upon dephosphorylation (50, 51). Given that cyclin L2 has multiple potential phosphorylation sites, especially in the RS domain, it is likely that other kinases or phosphorylation sites play a role in its phosphorylation in macrophages. This could explain the moderate increases in HIV replication when the phospho mutants were expressed in macrophages compared to the wild type.

Our previous studies showed cyclin L2-mediated degradation of SAMHD1 resulted in fewer HIV reverse transcription products. In this study, DYRK1A did not affect HIV reverse transcripts but knockdown increased HIV mRNA production. Therefore, it is possible that cyclin L2 affects the HIV life cycle at two distinct points depending on the specific interacting protein partner.

From the foregoing, a model emerges whereby cyclin L2 control of HIV replication in macrophages is modulated in part by the kinase DYRK1A. Interaction between the two proteins keeps the levels of cyclin L2 to a minimum, a situation that favors HIV restriction in macrophages. We conclude that regulation of cyclin L2 levels by DYRK1A contributes to HIV restriction in macrophages.

## MATERIALS AND METHODS

### Cell culture, reagents, and antibodies.

HeLa, 293T, and TZM-bl cells were maintained in Dulbecco’s modified Eagle’s medium (DMEM) supplemented with 10% fetal bovine serum (FBS), antibiotic-antimycotic, glutamine, and sodium pyruvate. THP-1 cells were maintained in RPMI medium supplemented with l-glutamine and 10% FBS, antibiotic-antimycotic, and sodium pyruvate. Where indicated, 50 ng/ml of phorbol 12-myristate 13-acetate (PMA) was used to differentiate THP-1 into macrophages for 48 h. HeLa, THP-1, and 293T cells were obtained from the ATCC. TZM-bl cells were obtained from the NIH AIDS Reagent Program, Division of AIDS, NIAID. Human monocyte-derived macrophages (MDMs) were prepared from HIV-1-negative donors using Ficoll-Hypaque density gradient centrifugation (GE Healthcare). Isolated monocytes were maintained in RPMI medium supplemented with 10% FBS and then differentiated with 50 ng/ml of macrophage colony-stimulating factor (M-CSF) for 7 days prior to transfection. Cyclin L2 CRISPR/Cas9 knockout in THP-1 cells was performed in collaboration with the Genome Engineering and iPSC Center (GEiC) at Washington University, targeting exons 2 and 6. The sequences of the guide RNA pairs were as follows: for exon 2, ACTTGGTATAAAAGAACCGCNGG and GTCCTTCGTGAAGCACTCCANGG, and for exon 6, CTTTGCCCAATCGTCCCCATNGG and AATGGGGACGATTGGGCAAANGG. The resulting cell line had no expression of cyclin L2 protein (see figures). HIV replication could be rescued with expression of cyclin L2 in these cells. Knockout THP-1 cells were maintained in RPMI medium as described above.

The 3-(4,5-dimethylthiazolyl-2)-2,5-diphenyltetrazolium bromide (MTT) assay reagents were obtained from Roche, and cell proliferation assays were performed according to the manufacturer’s protocol and as described previously (58). In these assays, MTT dye is reduced to formazan by mitochondrial enzymes from viable cells. Absorbance reading increases with cellular proliferation.

Goat polyclonal cyclin L2 and goat polyclonal actin primary antibodies were purchased from Santa Cruz Biotechnologies. Rabbit cyclin L2 polyclonal antibody was purchased from Novus Biological (number NB100-87009) and ProSci Inc. (number 8005). Myc antibody was obtained from Clontech. Donkey anti-goat secondary antibody was obtained from Santa Cruz Biotechnologies. Goat anti-mouse and goat anti-rabbit antibodies were purchased from Invitrogen. DYRK1A antibody was from Abnova (H00001859-M01).

### Plasmid, siRNA, and shRNA transfections.

Full-length Myc-cyclin L2 was purchased from Origene (RC213824). This was used as a template to make constructs of cyclin L2 by standard PCR and cloning methods. To express wild-type and mutant cyclin L2 in THP-1, full-length cyclin L2 (cyclin L2 WT), cyclin L2 P369 (S330A, S338A, S348A, and S369A), and cyclin L2 P330 (S330A, S338A, S348A, S369A, S394A, S401A, and S427A) were cloned into doxycycline-inducible lentiviral vector TtRMPVIR, which was a gift from Scott Lowe (Addgene; plasmid number 27995) (59). Mutants for cyclin L2 were made in wild-type cyclin L2 in pCMV6-Entry vector (Origene; RC213824) and cloned into TtRMPVIR vector (Addgene; 27995) using standard mutagenesis, PCR, and cloning methods.

A pool of four siRNAs (SMARTpool) for DYRK1A was obtained from Dharmacon (GE Healtcare; catalog number L-004805-00-0005). shRNAs for DYRK1A were obtained from the Washington University Genome Center. A cocktail of three shRNAs with sequences CCAGCAGTTGATTCTTGTATT, GCTGCTAATACCTTGGACTTT, and CAGTATATTCAGAGTCGCTTT was used. The HIV-1 *luc* Δ*env* reporter virus was derived from a pNL4-3 backbone, and the Nef gene replaced the luciferase gene. The NL4-3 lucmCherry virus was a gift from Warner Greene (12). HIV-1 BaL-3 virus, contributed by Bryan R. Cullen (60), was obtained from the AIDS Reagent Program. The NL4-3 lucmCherry and BaL-3 viruses have all HIV genes intact, including the Nef gene. Plasmids containing cyclin L2 constructs were used to transfect 293T cells and HeLa cells using a Polyplus jetPRIME kit according to the manufacturer’s protocol. For siRNA knockdown of DYRK1A in 293T, HeLa, and THP-1 cells and MDMs, cells were transfected by nucleoporation using Lonza Nucleofector according to the manufacturer’s protocol (61). After transfection, the cells were incubated for 48 h before infection with HIV-1Luc.

### Immunofluorescence.

HeLa cells expressing green fluorescent protein (GFP)-DYRK1A cultured on coverslips were rinsed once with phosphate-buffered saline (PBS), fixed for 10 min with 4% paraformaldehyde, then washed twice with 1× PBS, and permeabilized with 0.1% Triton X-100. Fixed cells were then blocked with 1% BSA in 1× PBS-Tween (PBST) at room temperature for 30 min, followed by overnight incubation with primary antibodies at 4°C. They were then washed with 1× PBST and incubated at room temperature for 1 h with Alexa Fluor secondary antibody. Control coverslips were prepared without primary antibodies. Images were taken and processed on a confocal fluorescence microscope.

### Cell lines, virus production, and infections.

Small hairpin RNA (shRNA) virus particles were obtained by cotransfecting 293Tcells using Lipofectamine 3000 reagent according to the manufacturer’s protocol (Life Technologies) with pLKO.puro vector containing DYRK1A cocktail shRNA, Gag-Pol, and vesicular stomatitis virus G protein (VSV-G) at a ratio of 10:2:1.

TtRMPVIR plasmids containing cyclin L2 WT, cyclin L2 P369, and cyclin L2 P330 were cotransfected in 293T cells with Gag-Pol and VSV-G plasmid at a ratio of 10:2:1. The viral particles were collected as supernatants 24 to 48 h posttransfection. Cleared supernatants were then used to transduce THP-1 cells to obtain stable cell lines for DYRK1A shRNA by puromycin selection, and cyclin L2 by GFP fluorescence activated sorting. HIV-BaL, HIV NL4-3, and HIV-1Luc were produced in 293T cells for 48 h and obtained as supernatants after transfection with Lipofectamine 3000 reagent according to the manufacturer’s protocol. Infection of 293T, HeLa, and THP-1 cells and MDMs was achieved by addition of 10 to 100 ng of p24 of virus to the cells in a 6- or 12-well plate depending on the cell type and the experiment. Differentiated THP-1 cells and monocyte-derived macrophages required higher concentrations of the virus. Cells were transduced for 6 h, washed once with PBS, and incubated further for 24 to 48 h. Firefly luciferase activity normalized to total protein concentration per well was then measured to assess viral replication postinfection. Reads of ≤300 U were considered background.

### Immunoprecipitations and Western blotting.

Twenty-four to 48 h posttransfection, cells were washed once with PBS and lysed with PBS buffer with 0.2% NP-40 and protease inhibitor cocktail (Roche). Total protein was measured with a bicinchoninic acid (BCA) protein assay kit. For immunoprecipitation assays, supernatants were incubated with immunoprecipitating antibody overnight at 4°C. Protein A- agarose beads were then used to capture the complexes for 2 h at room temperature or overnight at 4°C, followed by three washes with PBS–0.1% NP-40. Immunoprecipitants were eluted with SDS sample buffer at 100°C for 5 min. Protein samples were separated on a 12.5% SDS-PAGE gel and transferred to nitrocellulose. The membrane was blocked for 1 h at room temperature or overnight at 4°C in 5% milk or 4% BSA in PBS–0.05% Tween 20, followed by incubation with primary antibodies overnight at 4°C. The membrane was washed with PBS–0.05% Tween and probed with horseradish peroxidase (HRP)-conjugated secondary antibodies at room temperature for 1 h. The membranes were stained with Immobilon Western chemiluminescent HRP (Millipore) or Femto SuperSignal, and quantitation was processed with ImageJ software.

### Statistics.

Student’s *t* test was used for pairwise comparisons and one-way analysis of variance (ANOVA) with a follow-up Tukey multiple-comparison test for multiple comparisons. *P* values of <0.05 were considered significant. GraphPad Prism 9.00 software (GraphPad Software, Inc., La Jolla, CA) was used for calculations.
